# Association of METS-IR with incident hypertension in non-overweight adults based on a cohort study in Northeastern China

**DOI:** 10.1093/eurpub/ckac140

**Published:** 2022-09-26

**Authors:** Chengyin Xu, Guirong Song, Dongmei Hu, Guorong Li, Qigui Liu, Xiao Tang

**Affiliations:** Department of Health Statistics, School of Public Health, Dalian Medical University, Dalian City, Liaoning Province, People’s Republic of China; Department of Epidemiology and Health Statistics, Fudan University, Shanghai, People’s Republic of China; Department of Health Statistics, School of Public Health, Dalian Medical University, Dalian City, Liaoning Province, People’s Republic of China; Department of Health Statistics, School of Public Health, Dalian Medical University, Dalian City, Liaoning Province, People’s Republic of China; Department of Health Statistics, School of Public Health, Dalian Medical University, Dalian City, Liaoning Province, People’s Republic of China; Department of Health Statistics, School of Public Health, Dalian Medical University, Dalian City, Liaoning Province, People’s Republic of China; Department of Health Statistics, School of Public Health, Dalian Medical University, Dalian City, Liaoning Province, People’s Republic of China

## Abstract

**Background:**

Insulin resistance (IR) plays an important role in the progression of hypertension (HTN); therefore, early identification of IR is clinically important for preventing HTN. Our study aims to explore the relationship between the metabolic score for IR (METS-IR) and HTN in Chinese population who maintained non-overweight.

**Methods:**

A total of 4678 adults who underwent annual health check-up in our institution from 2010 to 2017, did not have HTN at the first check-up and maintained non-overweight at follow-up were selected as subjects. The baseline METS-IR was calculated and the outcome was incident HTN. Cox proportional hazards regression models were used to evaluate hazards ratios of HTN for METS-IR. Additionally, sensitive analyses and stratification analyses were used to deeply verify the relationship of METS-IR with HTN. The dose–response association between METS-IR and HTN risk was investigated using restricted the cubic spline analysis fitted for the Cox proportional hazards model.

**Results:**

Compared with the first quartiles of METS-IR, the risk of incident HTN was increased by 58% [hazard ratio (HR) 1.58, 95% confidence interval (CI) 1.12–2.22] and 96% (HR 1.96, 95% CI 1.40–2.76) in the Q3 group and the Q4 group, respectively. The results remained consistent when analyses were restricted to people without abnormal high-density lipoprotein cholesterol, triglyceride or fasting plasma glucose level at baseline. A linear dose–response relationship between METS-IR and HTN risk was identified (HR 1.08, 95% CI 1.04–1.12).

**Conclusions:**

The risk of incident HTN was associated with elevated METS-IR levels in non-overweight individuals. METS-IR could help predict the risk of HTN in non-overweight individuals.

## Introduction

Hypertension (HTN) is a major risk factor for cardiovascular disease (CVD) and chronic renal disease and has become a serious global public health issue.^1^^,^^2^ The prevalence of HTN in adults is above 27.9% in China, according to the National Hypertension Survey (China Hypertension Survey, CHS) in 2018. Therefore, about one in four adults is a patient with HTN and the total number of patients with HTN is 244 million in China.^3^ Due to a large population of HTN, early prediction and prevention of HTN is therefore an essential issue.

According to several studies, insulin resistance (IR) is the pathophysiological foundation of HTN and plays a significant role in its development and progression.^4^^,^^5^ As a result, early and precise identification of IR has important clinical significance for developing prevention strategies and optimal managements of HTN. Due to the complex operation, invasiveness and high cost, traditional IR evaluation methods including the hyperinsulinemic–euglycemic clamp test (HEC) and homeostasis model assessment-IR^6^^,^^7^ limit their practical utility. Metabolic score for IR (METS-IR) was a comprehensive evaluation index formed by body mass index (BMI), triglyceride (TG), blood glucose [fasting plasma glucose (FPG)] and high-density lipoprotein cholesterol (HDL-C), which was reported to offer higher concordance with EHC than other non-insulin-based IR indexes like TGs-to-HDL-C ratio (TG/HDL-C) and TG-glucose index recently.^8^ There has been few research on the association between the METS-IR and HTN.^9^^,^^10^ In a cross-sectional study of 142 005 participants from China, METS-IR was found to be linked with HTN only in people with a non-overweight BMI.^10^ Another study of community-dwelling older people (≥65 years) in China showed that METS-IR was substantially linked with HTN.^9^ However, to our best knowledge, cohort studies exploring the capacity of METS-IR to predict HTN are very scarce.^11^ Additionally, no cohort study has examined the association between METS-IR and incident HTN in a non-overweight population.

Metabolically obese normal weight (MONW) refers to an abnormal increase in metabolic parameters, but BMI within the normal range. Recent studies have shown that people with MONW are more likely to develop elevated IR levels, type 2 diabetes and HTN.^12^^,^^13^ According to a national representative investigation in China, the prevalence of HTN was found to be 15.4% in normal-weight people.^3^ Given that IR is a key pathogenic factor in the development of HTN,^14^ it is very beneficial to determine a simple and effective IR surrogate for early identification of high-risk HTN individuals in normal-weight people. Although some cross-sectional studies have suggested that METS-IR is linked to HTN in people with normal or lean weight,^10^^,^^11^ no longitudinal investigation has established that METS-IR is a predictor of HTN in people who have always maintained a non-overweight.

We intended to investigate the relationship between the METS-IR and incident HTN in people who maintained non-overweight at follow-up. To our knowledge, this was the first longitudinal cohort analysis of its kind to look at the link between METS-IR and incident HTN in non-overweight people.

## Methods

### Study participants

This study is based on data from participants aged 18–80 who underwent annual health check-ups at a hospital in Northeast China from 2010 to 2017. Their medical examination records included gender, age, medical history, medication history, family history, height, weight, blood lipids, blood glucose and blood pressure. The inclusion criteria at baseline were: (i) subjects without a medical history of HTN, diabetes, CVD, coronary heart disease, autoimmune liver disease, cirrhosis, viral hepatitis and renal tuberculosis at baseline. (ii) Subjects who were non-overweight at baseline. (iii) Subjects who did not take medicines that may affect blood pressure, blood glucose or blood lipids at baseline or follow-up. A total of 6877 individuals met the inclusion criteria. Subjects were excluded based on the following criteria: (i) subjects without complete data associated with METS-IR at the baseline. (ii) Subjects without follow-up visit records. (iii) Subjects who developed overweight or obese during follow-up. These limits left 4678 participants available for the final analysis.

The analysis was carried out in compliance with the Helsinki Declaration principles. The Dalian Medical University Ethics Committee approved the study procedure (Ethics Approval No. 2020006). The ethical committee decided to waive the study’s informed consent requirement because we eliminated all identifiable personal information.

### Measurement

When participants stood erect and barefoot, their height was measured to the closest 0.5 cm. When participants wore light-weight clothing, their weight was measured to the closest 0.5 kg. BMI was defined as the weight (kilograms) by the square of height (meters). After resting for more than 5 min, a typical electronic sphygmomanometer was used to monitor sitting blood pressure on the right upper arm. If the blood pressure was abnormal, the participant was advised to remeasure after a 20-min’ rest, and the lower one was read as the final blood pressure.

Participants were asked to fast for at least 12 h before the check-ups and to avoid smoking, alcohol and caffeine. Biochemical measures including FPG, TG, total cholesterol (TC), low-density lipoprotein cholesterol (LDL-C), HDL-C, aspartate transferase (AST) and alanine transferase (ALT) were measured on a Siemens ADVIA 2400 automatic biochemical analyzer.

### Definitions

HTN was defined as systolic blood pressure (SBP) ≥140 mmHg, diastolic blood pressure (DBP) ≥90 mmHg or undergoing HTN therapy.^3^ METS-IR was calculated as (ln [(2 × FPG) + TG)] × BMI)/(ln [HDL-C]) (FPG, TG and HDL-C levels were expressed as mg/dl and BMI as kg/m^2^ in the equation).^8^ Overweight was defined as BMI ≥24 kg/m^2^, and non-overweight was defined as BMI <24 kg/m^2^.

### Statistical analysis

Continuous data were presented as mean and standard deviation, and categorical variables as frequency and percentage. The Chi-square test was performed to compare categorical variables, and the two independent sample *t*-test was utilized to examine the difference of continuous variables by HTN status. Baseline METS-IR was divided into four quartiles as follows: Q1 (≤26.80), Q2 (26.81–29.18), Q3 (29.19–31.90) and Q4 (≥31.91). One-way analysis of variance was used to compare the participants’ baseline features among the METS-IR quartiles.

The Kaplan–Meier analysis was used to assess the cumulative hazards of HTN among METS-IR quartiles, and the differences of curves were examined utilizing the log-rank test. Cox proportional-hazards regression models were used to evaluate the risk of HTN for each METS-IR quartile, setting the first quintile of METS-IR as the reference group. Model 1 was adjusted for age and sex, and model 2 was further adjusted for TC, LDL-C, ALT and AST. The dose–response relationship between METS-IR and the risk of HTN was investigated through restricted cubic splines (RCS) analysis, with three knots at the METS-IR’s 1st, 50th and 90th percentiles. The reference point in the spline model was the median value of the 1st quartile of the METS-IR (25.12), and the confounding variables were the same as in model 2.^15^

Participants with FPG ≥7.0 mmol/l, HDL-C ≤ 1.0 mmol/l and TG ≥1.7 mmol/l, at baseline were excluded from sensitivity analysis to ensure the results were robust. The model in the sensitivity analysis was also adjusted for confounding factors same as those in model 2. Additionally, considering that different basic demographic characteristics (age and gender) and different traditional cardiovascular and cerebrovascular indicators (LDL-C and TC) levels have different effects on HTN, subgroup analyses were performed to explore the relationship between METS-IR and incident HTN by sex, age (<40 or ≥40 years), TC (<200 or ≥200 mg/dl) and LDL-C (<130 or ≥130 mg/dl).^16^

R 4.0.3 (http://www.Rproject.org) was used for the RCS analysis, and SPSS Statistic version 25.0 was used for all other statistical analysis (Chicago, IL, USA).

## Results

### Baseline characteristics of study participants

The study included 4678 non-overweight adults aged 18–80 years at research entry, with a mean follow-up period of 3.00 years. After 13 876.75 person-years of follow-up, 403 subjects (173 males and 226 females) developed HTN, and the incidence density of HTN was 26.40 per 1000 person-years.

The subjects’ features at baseline were shown in table 1 according to the development of HTN. Baseline age, METS-IR, SBP, FPG, BMI, TC, TG, LDL-C, AST and ALT were significantly higher in subjects with HTN than in those without HTN. The typical baseline characteristics among METS-IR quartiles are shown in Supplementary table S1. Across increasing quartiles, an increased percentage of male participants as well as increased levels of age, SBP, DBP, BMI, TG, TC, LDL-C, HDL-C, FPG, AST and ALT had been observed (All *P*_trend_ < 0.001).

**Table 1:** Baseline characteristics of study population by HTN status at follow-up

Characteristics	Overall	Non-HTN	HTN	*P* value
*N*	4678	4275	403	<0.001
Age, years	35.8 ± 10.1	35.2 ± 9.6	42.1 ± 12.4	<0.001
Male	1179 (25.2)	1006 (23.5)	173 (42.9)	<0.001
METS-IR	29.4 ± 3.7	29.3 ± 3.7	30.9 ± 3.8	<0.001
BMI, kg/m^2^	20.9 ± 1.7	20.9 ± 1.7	21.5 ± 1.6	<0.001
SBP, mmHg	115.0 ± 11.5	114.0 ± 11.2	125.0 ± 9.9	<0.001
DBP, mmHg	69.9 ± 8.6	69.2 ± 8.4	76.6 ± 8.3	<0.001
FPG, mg/dl	94.5 ± 9.3	94.2 ± 9.2	97.3 ± 9.8	<0.001
TG, mg/dl	82.6 ± 42.1	80.9 ± 40.7	99.8 ± 50.8	<0.001
TC, mg/dl	172.2 ± 26.7	171.4 ± 26.2	180.0 ± 30.3	<0.001
HDL-C, mg/dl	55.1 ± 11.4	55.7 ± 11.5	53.4 ± 10.9	<0.001
LDL-C, mg/dl	99.4 ± 27.3	97.2 ± 26.4	108.6 ± 30.2	<0.001
ALT, U/l	17.9 ± 11.8	17.6 ± 11.7	21.1 ± 12.7	<0.001
AST, U/l	20.4 ± 6.8	20.2 ± 6.6	22.4 ± 8.1	<0.001

Data are the mean ± standard deviation or number (percentage).

METS-IR, metabolic score for insulin resistance; BMI, body mass index; SBP, systolic blood pressure; DBP, diastolic blood pressure; FPG, fasting plasma glucose; TG, triglycerides; TC, total cholesterol; HDL-C, high-density lipoprotein cholesterol; LDL-C, low-density lipoprotein cholesterol; ALT, alanine transferase; AST, aspartate transferase.

### The association between METS-IR and incident HTN

**Figure 1:**
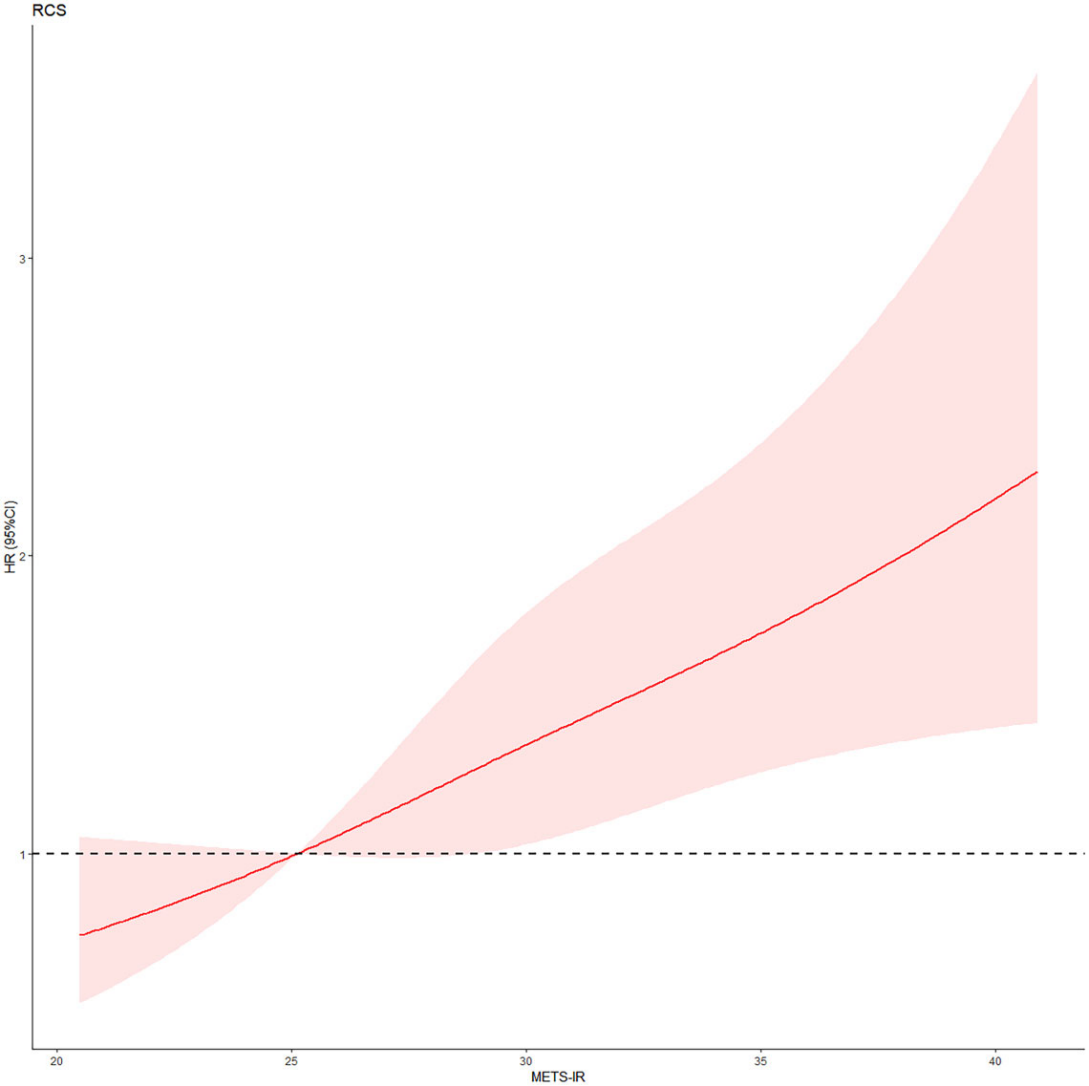
Cumulative hazard ratio for incident HTN by quartiles of METS-IR

**Figure 2:**
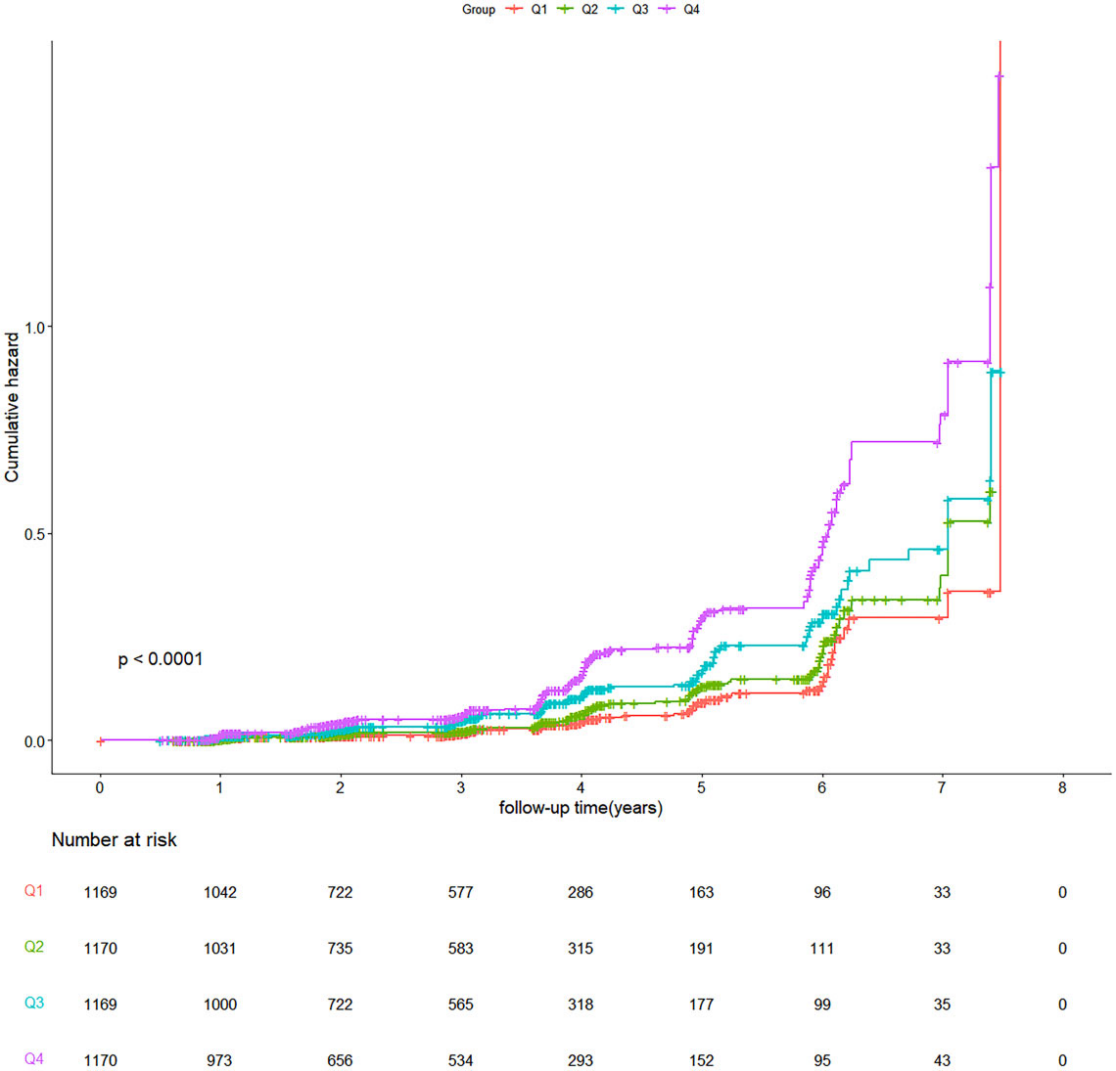
Dose–response relationship between METS-IR and HTN. Model was adjusted for age, sex, ALT, AST, TC and LDL-C

The cumulative hazard of HTN increased with increasing quartiles (figure 1). The effect sizes of the METS-IR, including hazard ratio (HR) and 95% confidence intervals (CIs), are presented in table 2. In the crude model, compared to the reference group (Q1), the risk of incident HTN increased by 0.95-fold (HR 1.95, 95% CI 1.41–2.71) in Q3 and by 2.05-fold (HR 3.05, 95% CI 2.24–4.16) in Q4, respectively. After adjusting for sex and age, the significant results were still observed in Q3 and Q4. After further full adjustment for confounders ALT, AST, TC and LDL-C in model 2, higher METS-IR quartile groups (Q3 and Q4) also presented with a higher risk of HTN (HR 1.58, 95% CI 1.12–2.22, and HR 1.96, 95% CI 1.40–2.76, respectively) when compared with the lowest quartile group (Q1). In addition, a one-unit rise in the METS-IR raised incident HTN’s probability by 14% in the crude model, and by 9% and 8% in models 1 and model 2, respectively (table 2). HR in all models showed a significant linear trend (*P*_trend_ < 0.05), RCS analysis indicated that there existed a linear dose–response link between METS-IR and the risk of HTN (*P*_non-linear_ = 0.6814, figure 2).

**Table 2:** The association between the risk of incident HTN and METS-IR

Variables	Crude	*P* value	Model 1^a^	*P* value	Model 2^b^	*P* value
	HR (95% CI)		HR (95% CI)		HR (95% CI)	
METS-IR (continuous)	1.14 (1.11, 1.18)	<0.001	1.09 (1.05, 1.13)	<0.001	1.08 (1.04, 1.12)	<0.001
METS-IR by quartiles						
Q1	1		1		1	
Q2	1.36 (0.96, 1.93)	0.087	1.27 (0.90, 1.81)	0.177	1.25 (0.88, 1.78)	0.216
Q3	1.95 (1.41, 2.71)	<0.001	1.60 (1.15, 2.23)	0.005	1.58 (1.12, 2.22)	0.009
Q4	3.05 (2.24, 4.16)	<0.001	2.10 (1.52, 2.88)	<0.001	1.96 (1.40, 2.76)	<0.001
*P*_trend_	<0.001		<0.001		<0.001	

a Model 1: adjusted for age, sex.

b Model 2: adjusted for age, sex, ALT, AST, TC and LDL-C.

### Sensitivity analysis

The sensitivity analyses restrained to subjects with normal TG, HDL-C and FPG also acquired significant results (Supplementary table S2). If the METS-IR increased by one unit, the risk of incident HTN increased by 9%. In addition, the sensitivity analysis showed that the risk of developing HTN was 0.47 and 1.08 times higher among participants with METS-IR in Q3 and Q4 than Q1, respectively. This result indicated that even individuals with normal TG, HDL-C, FPG and BMI have an expanded the risk of subsequent HTN due to elevated levels of METS-IR.

### Subgroup analysis

The effects of METS-IR on incident HTN in subgroups are presented in Supplementary table S3. After adjusting for age, sex, AST, ALT, LDL-C and TC, METS-IR remained a significant predictor of incident HTN in the six subgroups of participants who were male, female, young (<40 years), old (≥40 years), with normal LDL-C (<130 mg/dl) and with normal TC (<200 mg/dl). The results in most subgroups were consistent with those in the whole population. In particular, METS-IR showed a strong relationship with incident HTN in the older group (≥40 years), in which HR was significant in each quartile, which indicated that METS-IR might a good predictor of HTN for older people.

## Discussion

### Main findings

This cohort study indicated the METS-IR is a good measure for identifying non-overweight adults who are at hazard of evolving HTN. METS-IR and the risk of HTN kept a linear dose–response relationship. The results remained consistent when the research was confined to members with normal TG, FPG and HDL-C at baseline. After stratification by age, sex and LDL-C levels, METS-IR remained a significant predictor of HTN occurrence in both males and females, in the older group (≥40 years), in the younger group (<40 years) and in the normal LDL-C group. To our knowledge, this is the first longitudinal cohort research to assess the link between the baseline METS-IR and incident HTN in non-overweight persons.

A considerable relationship between METS-IR and the risk of HTN has been newly indicated by some prospective and cross-sectional researches.^9^^,^^11^ A cross-sectional survey of 4352 Chinese older adults showed that after adjusting for gender, age, alcohol expenditure, smoking, physical activity and education, METS-IR was significantly associated with HTN, which was partly consistent with our results. However, this research only contained older adults (≥65 years).^9^ A prospective cohort research in Mexico with an average follow-up time of 2.4 years reported that METS-IR was considerably higher in patients with HTN than in participants without HTN at baseline. And compared with people in the lowest METS-IR group, the risk of HTN was 81% higher in those with METS-IR in the highest tertile after adjustment for known cardiovascular risk factors, which result was same as our findings.^11^ However, this study included overweight and obese people, so the effects of overweight and obesity on IR cannot be ruled out. Additionally, a cross-sectional research including 142 005 Chinese participants who did not accept antihypertensive medication demonstrated that METS-IR was related with HTN considerably only in individuals with normal BMI.^10^ It might be due to the absence of subcutaneous fat in people with lean body mass, making them more susceptible to ectopic fat storage in the muscle and liver and hypertriglyceridemia, which results in impaired metabolism.^17^ We conducted a cohort study of people that were aged 18–80 years maintaining normal weight and confirmed that METS-IR was related with HTN significantly, so it is expected that METS-IR will play a role in short-term HTN hazard management.

Several investigations show that IR is the general pathophysiological basis of prediabetes and prehypertension.^8^^,^^18^ IR can increase blood pressure through various mechanisms such as changing sympathetic nervous system activity, enhancing renal sodium and water retention, and increasing vasoactive substances. The METS-IR formula’s each component is connected with HTN's evolution. BMI is closely related to the pathological mechanism of HTN, therefore as a recognized predictive indicator of HTN.^19^^,^^20^ Increased BMI indicates excess adipose tissue (especially visceral adipose tissue) in the body, causing increased free fatty acid (FFA) discharge and continuous increase of inflammatory mediators like IL-6 and tumor necrosis factor α, which are closely related to IR caused by impaired lipid homeostasis and inflammatory response.^21^^,^^22^ Apart from BMI, it is considerably reported that dyslipidemia, including elevated TG, low HDL-C and elevated LDL-C were linked with HTN independently.^23^^,^^24^ Lower HDL-C level leads to reduced release of vasodilator nitric oxide and promotes adhesion of monocytes to vascular endothelial cells, both of which can increase peripheral vascular resistance and increase blood pressure.^25^ On the other hand, nitric oxide’s output is inhibited by the rise of TG and LDL-C, thus promoting vascular endothelial disorder and leading to elevated blood pressure.^26^ Fasting TG level mainly represents IR of adipose cells.^27^ FFAs and diglycerol are metabolites of TG, which can inhibit insulin receptors and substrates by activating various serine and threonine kinases, thereby regulating the insulin signaling pathway and ultimately inducing peripheral IR in the body.^28^ And then, insulin-mediated smooth muscle cell growth and proliferation are enhanced, leading to increased blood pressure.^29^ FPG level, another component of METS-IR, represents hepatic insulin performance primarily. In the condition of fasting and IR in the liver, the rate of endogenous glucose synthesis increases, and the glucose and lipid metabolism was disturbed, and then higher insulin concentration enhances the sodium’s reabsorption in renal tubules, leading to sodium and water retention, lifted blood volume and increased blood pressure.^25^^,^^30^ All of these might explain why the combined monitoring indicator of METS-IR helps develop strategies for the primary prevention and management of HTN.^10^^,^^11^^,^^31^

The results of the sensitivity analysis in this study suggested that METS-IR might increase the effectiveness of prediction of HTN in normal-weight people, as METS-IR was related with HTN meaningfully even in subjects with normal HDL-C, FPG, TG and BMI levels. The mechanism might be related to the diverse influence of IR on glycolipid metabolism and blood pressure. Compensatory hyperinsulinemia in the IR’s prophase might trigger vascular smooth muscle cell proliferation, and then lead to vascular stiffness and blood pressure rise.^32^ Furthermore, IR activates the sympathetic nervous system and the renin–angiotensin–aldosterone system,^33^ which can cause a rapid rise in blood pressure. When IR develops, unlike blood pressure elevation, FPG may continue to be normal for a duration of time on account of compensatory hyperinsulinemia.^34^ FPG may eventually grow greatly when b-cells are unable to react to IR and are unable to tolerate hyperinsulinemia, but this process takes a long time.^35^ Consequently, high blood pressure might be an early sign of IR, and METS-IR can be a better predictor of HTN than a single indicator in the early stages of IR. Clinicians should also pay attention to individuals with normal FPG, HDL-C or TG when developing HTN risk management strategies.

Our results suggested that persistently elevated METS-IR levels indicated an increased risk of HTN in non-overweight people, so we should not focus solely on changes in a single indicator, but rather promote a focus on comprehensive effect of FPG, BMI, TG and HDL-C. When an increase in METS-IR levels or adverse change in these four indicators is observed in clinical practice, physicians should remind people to control their weight, regulate the homeostasis of blood glucose and lipids through healthy lifestyle and behaviors such as adequate sleep, moderate exercise, and healthy diet,^36–38^ even in non-overweight people who do not have the most traditional risk factors for HTN.

The key strengths of this study were the longitudinal study and exploration of non-overweight people throughout follow-up. In the longitudinal research, it was clearer to verify the effectiveness of METS-IR on incident HTN. Furthermore, our research eliminated the effect of BMI on HTN and pointed the way to prevention for non-obese patients, who are more prone to be neglected.

### Limitations

There are several limitations to this study. First, insulin metrics was not measured by us directly in the research population, so we could not compare METS-IR’s predictive capability with straightforward markers of IR for HTN risk. Second, individuals’ information on lifestyle factors like physical exercise, calorie intake, alcohol intake and smoking status were unavailable, all of which might affect blood pressure. However, biomarkers such as ALT, AST, TC and LDL-C, which are heavily impacted by individual’s behavior, were included in our analysis. And that could have attenuated the effect of information of personal lifestyle on this study. Third, the subjects were from a certain health check-up center, and the sample size was not large enough, which might cause some bias. Large and representative epidemiological studies are still needed in the future. Fourth, the information in this study was obtained from Chinese adults, so it is unsure whether the discoveries can apply to other ethnic groups.

## Conclusions

In conclusion, our study’s results suggested that METS-IR was a reliable predictor for incident HTN in non-overweight Chinese adults. We therefore proposed that METS-IR was a cost-effective and uncomplicated index that can be practiced for the management and prevention of HTN, particularly for non-overweight people.

## Supplementary data

Supplementary data are available at *EURPUB* online.

## Supplementary Material

ckac140_Supplementary_Data

## Data Availability

The data underlying this article will be shared on reasonable request to the corresponding author.
