# Supplementary material for: Association of METS-IR with incident hypertension in non-overweight adults based on a cohort study in Northeastern China
Source: Eur J Public Health. 2022 Sep 26;32(6):884–90. doi: 10.1093/eurpub/ckac140 (PMC9713393; doi:10.1093/eurpub/ckac140)
Supplement: ckac140_Supplementary_Data [file ckac140_supplementary_data.docx]

**Table S1: Baseline characteristics of study population by quartiles of METS-IR**

| Characteristics | METS-IR quartiles | | | | P | P _trend_ |
| --- | --- | --- | --- | --- | --- | --- |
|  | Q1 | Q2 | Q3 | Q4 |  |  |
| N | 1169 (25.0) | 1170 (25.0) | 1169 (25.0) | 1170 (25.0) |  |  |
| Age, years | 32.0 ± 8.8 | 34.5 ± 9.3 | 37.1 ± 10.0 | 39.7 ± 10.6 | <0.001 | <0.001 |
| Male | 127 (10.9) | 181 (15.5) | 304 (26.0) | 567 (48.5) | <0.001 | <0.001 |
| BMI, kg/m^2^ | 18.8 ± 1.2 | 20.6 ± 0.9 | 21.7 ± 0.9 | 22.6 ± 0.9 | <0.001 | <0.001 |
| SBP, mmHg | 112.3 ± 11.0 | 114.3 ± 11.4 | 115.4 ± 11.1 | 118.1 ± 11.7 | <0.001 | <0.001 |
| DBP, mmHg | 68.7 ± 8.3 | 69.0 ± 8.5 | 69.7 ± 8.6 | 72.0 ± 8.7 | <0.001 | <0.001 |
| FPG, mg/dL | 91.1 ± 7.3 | 92.7 ± 7.4 | 95.5 ± 8.0 | 98.7 ± 11.7 | <0.001 | <0.001 |
| TG, mg/dL | 61.0 ± 20.0 | 69.9 ± 25.4 | 80.2 ± 31.4 | 118.0 ± 55.7 | <0.001 | <0.001 |
| TC, mg/dL | 170.3 ± 24.9 | 171.5 ± 26.5 | 171.9 ± 26.3 | 174.9 ± 28.7 | <0.001 | <0.001 |
| HDL-C, mg/dL | 63.6 ± 10.1 | 58.9 ± 9.6 | 53.8 ± 8.2 | 44.8 ± 7.3 | <0.001 | <0.001 |
| LDL-C, mg/dL | 88.9 ± 24.4 | 96.1 ± 25.9 | 100.44 ± 26.4 | 107.4 ± 27.7 | <0.001 | <0.001 |
| ALT, U/L | 15.3 ± 11.6 | 16.5 ± 9.6 | 17.9 ± 11.5 | 21.9 ± 13.2 | <0.001 | <0.001 |
| AST, U/L | 19.8 ± 6.9 | 20.0 ± 6.3 | 20.1 ± 7.0 | 21.4 ± 6.8 | <0.001 | <0.001 |

Data are the mean ± standard deviation or number (percentage)

Abbreviations: METS-IR, metabolic score for insulin resistance; BMI, body mass index; SBP, systolic blood pressure; DBP, diastolic blood pressure; FPG, fasting plasma glucose; TG, Triglycerides; TC, total cholesterol; HDL-C, high-density lipoprotein cholesterol; LDL-C, low-density lipoprotein cholesterol; ALT, alanine transferase; AST, aspartate transferase.

**Table S2 Sensitivity analyses for association of METS-IR with incident HTN**

|  | METS-IR (continuous) | METS-IR quartiles | | | | P trend |
| --- | --- | --- | --- | --- | --- | --- |
|  |  | Q1 | Q2 | Q3 | Q4 |  |
| Case, n | 4090 | 1162 | 1140 | 1086 | 702 |  |
| No. of HTN | 322 | 52 | 74 | 97 | 99 |  |
| No. of person-years | 12184.58 | 3452.97 | 3478.41 | 3247.18 | 2006.01 |  |
| Incidence density^a^ | 26.4 | 15.1 | 21.3 | 29.9 | 49.4 | 0.030 |
| HR | 1.09 (1.05, 1.14) | 1 | 1.23 (0.86, 1.76) | 1.47 (1.03, 2.10) | 2.08 (1.44, 3.01) | <0.001 |
| P | <0.001 |  | 0.258 | 0.031 | <0.001 |  |

^a^: per 1000 person-years.

Restricted analysis was excluded the participants with abnormal FBG (≥ 7.0 mmol/L), abnormal TG level (≥ 1.7 mmol/L), and abnormal HDL-C level (≤ 1.0 mmol/L) at baseline (n=588).

The model was adjusted for age, sex, ALT, AST, TC, and LDL-C.

**Table S3 The association of METS-IR with incident HTN in the subgroup analysis**

| Subgroup | Case, n | METS-IR (continuous) | METS-IR quartiles | | | |
| --- | --- | --- | --- | --- | --- | --- |
|  |  |  | Q1 | Q2 | Q3 | Q4 |
| **Sex** |  |  |  |  |  |  |
| Male | 1179 | 1.10 (1.04, 1.16) | 1 | 2.23 (1.03, 4.85) | 3.14 (1.52, 6.48) | 3.19 (1.57, 6.50) |
| Female | 3499 | 1.06 (1.01, 1.04) | 1 | 1.01 (0.67, 1.51) | 1.12 (0.73, 1.69) | 1.63 (1.07, 2.48) |
| **Age, years** |  |  |  |  |  |  |
| < 40 | 1639 | 1.07 (1.02, 1.14) | 1 | 0.81 (0.51, 1.29) | 1.14 (0.72, 1.81) | 1.72 (1.08, 2.75) |
| ≥ 40 | 3039 | 1.10 (1.05, 1.16) | 1 | 3.02 (1.55, 5.89) | 3.49 (1.82, 6.69) | 3.88 (2.04, 7.39) |
| **LDL-C, mg/dl** |  |  |  |  |  |  |
| Normal (< 130) | 4150 | 1.10 (1.06, 1.15) | 1 | 1.23 (0.84, 1.81) | 1.48 (1.02, 2.15) | 2.25 (1.55, 3.28) |
| Abnormal (≥ 130) | 528 | 1.02 (0.94, 1.10) | 1 | 1.68 (0.60, 4.69) | 2.39 (0.89, 6.40) | 1.64 (0.62, 4.36) |
| **TC, mg/dl** |  |  |  |  |  |  |
| Normal (< 200) | 4234 | 1.09 (1.04, 1.13) | 1 | 1.14 (0.79, 1.66) | 1.43 (1.00, 2.05) | 1.95 (1.36, 2.80) |
| Abnormal (≥ 200) | 444 | 1.08 (0.99, 1.18) | 1 | 3.49 (0.96, 12.71) | 4.33 (1.25, 15.06) | 3.84 (1.13,13.05) |

The model was adjusted for age, sex, ALT, AST, TC, LDL-C.
